# The process of recovery of people with mental illness: The perspectives of patients, family members and care providers: Part 1

**DOI:** 10.1186/1472-6963-10-161

**Published:** 2010-06-11

**Authors:** Sylvie Noiseux, Denise Tribble St-Cyr, Ellen Corin, Pierre-Luc St-Hilaire, Raymond Morissette, Claude Leclerc, Danielle Fleury, Luc Vigneault, Francine Gagnier

**Affiliations:** 1 Research Centre of the University of Montreal Hospital Centre (CR-CHUM), Masson Building, 3850 Saint Urbain Street, Montreal, Quebec, H2W 1T7, Canada; 2 Faculty of Nursing, University of Montreal, P.O.B. 6128, Centre-Ville Station, Montreal, Quebec, H3C 3J7, Canada; 3 School of Nursing Sciences, Faculty of Medicine and Health Sciences, Sherbrooke University, 3001, 12th Avenue North, Sherbrooke, Quebec, J1 H 5N4, Canada; 4 Sherbrooke University Hospital Research Centre (CRC-Etienne-Lebel-CHUS), Canada; 5 Douglas Research Institute, Lehmann Building, 6875 LaSalle Boulevard, Suite G-1123, Verdun, Quebec, H4 H 1R3, Canada; 6 Research Centre of the University of Montreal Hospital Centre (CR-CHUM), Masson Building, 3850 Saint Urbain Street, Montreal, Quebec, H2W 1T7, Canada; 7 Louis-H. Lafontaine Hospital, 7401 Hochelaga Street, Montreal, Quebec, H1N 3M5, Canada; 8 Department of Nursing Sciences, University of Quebec at Trois-Rivières, 3351 Des Forges Boulevard, Trois-Rivières, Quebec, G9A 5H7, Canada; 9 Director of Nursing, University of Montreal Hospital Centre (CHUM), 1001 Saint Denis Street, Montreal, Quebec, H2X 3H1, Canada; 10 Executive Director, Association des personnes utilisatrices des services de santé mentale, Quebec Region (APUR), 165 Carillon Street, Suite 316, Quebec, Quebec, GIK 9E9, Canada; 11 Systems Analyst, University of Montreal, 7077 Du Parc Avenue, Suite 3035, Montreal, Quebec, H3N 1X7, Canada

## Abstract

**Background:**

It is a qualitative design study that examines points of divergence and convergence in the perspectives on recovery of 36 participants or 12 triads. Each triad comprising a patient, a family member/friend, a care provider and documents the procedural, analytic of triangulating perspectives as a means of understanding the recovery process which is illustrated by four case studies. Variations are considered as they relate to individual characteristics, type of participant (patient, family, member/friend and care provider), and mental illness. This paper which is part of a larger study and is based on a qualitative research design documents the process of recovery of people with mental illness: Developing a Model of Recovery in Mental Health: A middle range theory.

****Methods**:**

Data were collected in field notes through semi-structured interviews based on three interview guides (one for patients, one for family members/friends, and one for caregivers). Cross analysis and triangulation methods were used to analyse the areas of convergence and divergence on the recovery process of all triads.

**Results:**

In general, with the 36 participants united in 12 triads, two themes emerge from the cross-analysis process or triangulation of data sources (12 triads analysis in 12 cases studies). Two themes emerge from the analysis process of the content of 36 interviews with participants: (1) *Revealing dynamic context*, situating patients in their dynamic context; and (2) *Relationship issues in a recovery process*, furthering our understanding of such issues. We provide four case studies examples (among 12 cases studies) to illustrate the variations in the way recovery is perceived, interpreted and expressed in relation to the different contexts of interaction.

**Conclusion:**

The perspectives of the three participants (patients, family members/friends and care providers) suggest that recovery depends on constructing meaning around mental illness experiences and that the process is based on each person's dynamic context (e.g., social network, relationship), life experiences and other social determinants (e.g., symptoms, environment). The findings of this study add to existing knowledge about the determinants of the recovery of persons suffering with a mental illness and significant other utilizing public mental health services in Montreal, Canada.

## Background

### Statement of the problem

The recovery process has become the guiding principle of the mental health system, resulting in advocacy for care and services that would facilitate the process and eliciting a clear political will and great enthusiasm in Canada [1,2]. However, recovery assumes numerous meanings, depending on the context in which it is raised, and the notion may create confusion among patients and their family as well as among clinicians, policy makers and researchers [3,4]. One may therefore ask: "How can one offer recovery-oriented services and care to people with a mental health disorder when the nature of recovery remains obscure or is often confused with concepts of remission, cure, readjustment, and even rehabilitation?"

Over the past twenty years, many research studies have indicated that recovery is not a cure but a profoundly personal path that individuals may follow; it entails work, particularly work on themselves, their feelings, desires, competencies, roles, and plans [4-7]. These studies shed light on the conditions associated with recovery but have not explained how the conditions affect each other or how the mechanisms by which they operate might help us understand the process by which people recover [3]. These limitations stem mainly from the research methods adopted and from the use of limited samples drawn from a single data source (e.g., patients) [8-11]. However, since family members/friends have known the individual involved since before the onset of the disease, their perceptions can enhance our understanding of the recovery process. In addition to that, recovery is not a purely individual process. It also has an inter-subjective character and involves the negotiation of the patient's place within a relational field. The perspectives of care providers are also important because they often witness significant outcomes in the condition of such individuals that go far beyond notions of stabilization [2,3]. However, apart from a few studies in which the investigators sought to verify how recent implementation of programs in the community may have fostered recovery [12-14], studies on the process have not given much consideration to the views of care providers. Furthermore, the term "recovery" is generally used for all serious mental health problems without distinction. Does that mean that recovery is the same for all people regardless of their different types of mental illness? It is clearly appropriate to broaden the study of recovery to include other diagnoses; mental health problems do present certain convergences, notably in terms of illness progression, but they differ in terms of the nature of the symptoms, the patient's experience of self others, and the world, and their impact on biopsychosocial functioning [2]. It is therefore important first to identify how the recovery process is perceived and negotiated between the different actors, and second, to identify the points of convergence and divergence which characterize the recovery of the persons specific problems of mental health, in order to develop the parameters of a theoretical model that would be general enough to orient the practitioners' gaze, and specific enough to take into account the singularity of the persons and their condition of health [2].

### Objective of the study

The objective of the study is to analyze the characteristic points of convergence and divergence between the three types of participants (patient, family member/friend, care provider) regarding the process of recovery for people with different kinds of mental illnesses.

### Research question

More specifically, the following question was addressed in this paper:

• What are the points of convergence and divergence between the three types of participants regarding the process of recovery for people living with mental illness?

## Methods

### Design

Given our current state of knowledge and the research question posed in this study, the methodology chosen was the case studies approach [15]. A qualitative case-study inductive design was selected to advance our understanding of the process of recovery in mental health [15-17]. The decision to collect and systematically compare data collected from various categories of participants was based on previous research done by Corin and associates [18-20].

By giving prominence to the perspectives of different groups of participants, qualitative research opens the way to learning--from the inside--about the dilemmas and issues people face in their recovery process. The decisions regarding the sample size of this theoretical sample (n = 36) of this case study are based on the criteria for qualitative research [17]. One of the principal criteria that the quantity and breadth of data gathered is more important than the number of participants. Indeed, in this study the triangulation of multiple sources of data (types of participants, four types of mental health problems); research tools (three interview guides, three sociodemographics questionnaires); one region with a large dense population (Montreal), together ensure a large quantity of content, the rigour of the process and provide strong substantiation of its constructs. Another factor influencing the decision on sample size was that it be adequate for achieving category saturation, not necessarily statistically representative. It is based rather on the information that appears necessary to reach empirical saturation, that is, the point at which participants add no new information about the phenomenon being studied. Moreover, constructing the sample in this way makes it possible to organize a complex universe and thus take a crucial step towards developing a substantive theoretical model [21]. In short, the methodological approach used allows us to triangulate the various perspectives. This study was inspired by the results obtained and tools developed in other studies of recovery conducted by the present group of investigators, which provided them with methodological experience to support their choices [3,4].

### Sampling Strategy and Description of Participants

The convenience sample consisted of 36 participants united in 12 triads. Each triad comprising a people with a mental health problem (schizophrenia, affective, anxiety, and borderline personality disorders), a family member/friend, and a care provider and documents the procedural, analytic and structural feasibility and acceptability of "triangulating" perspectives as a means of understanding the recovery process (see Table 1). Participants were be selected in accordance with the following inclusion and exclusion criteria: To be included in the study, people with a mental health problem must: (a) have a diagnosis of schizophrenia disorder, affective disorder, anxiety disorder, or borderline personality disorder based on a psychiatric evaluation; (b) be able to identify a significant family member and a care provider likely to take part in the study; (c) live in the community; (d) be in a stable condition (able to manage symptoms) and (e) know how to speak, read and write French. To be included, family members must: (a) have a significant bond with the person with the mental health problem who has agreed to take part in the study (e.g., parent, spouse, brother, sister, friend) and (b) know how to speak, read and write French. To be included, care providers must: (a) have followed a person with a mental health problem who has agreed to take part in the study for at least the past year (nurse, psychiatrist, social worker, psychologist, etc.) and (b) know how to speak, read and write French. People with a mental health problem and their family members are excluded if they: (a) go through an acute crisis episode and are hospitalized; (b) have a major physical health problem; or (c) are diagnosed as intellectually deficient.

**Table 1:** Formation of the theoretical sample in Montreal

	Types of mental health problems				
**Site**	**Schizophrenia**	**Affective**	**Anxiety**	**Borderline personality**	**Total**

Montreal	3T	3T	3T	3T	12T

Total	n = 9	n = 9	n = 9	n = 9	N = 36

**1 T **= 1 person with a mental health problem, 1 family member or friend and 1 care provider

The sample comes from French districts in the area of Montreal, Canada: two types of settings were selected in order to include a variety of participants or actors (patients, family members or friends, care providers) and data sources (structured treatment programs administered by a hospital, community organizations). Due to its composition, the sample represents a unique opportunity for collecting a wide range of data that will make it possible to compare the perspectives of three participants who, in one way or another, are involved in an experience of recovery.

### Sociodemographic Characteristic's of Patients, Family Members/Friends and Care providers

The patients included two men and ten women. Their mean age was 38 years. Eight of them were employed, ten lived alone, and two were in common law relationships. At the time of the interview, all were taking a psychotropic medication. The mean age of the three men and nine women who made up the sample of family members and friends was 49 years. Six were family members (mother, father, and daughter), four were friends, and two were spouses. The care providers--three men and nine women-included two psychologists, four nurses, two social workers, two occupational therapists, and two community organization support workers. They had a mean 14 years of experience in the field of psychiatry (see Table 2 ).

**Table 2:** Participant's sociodemographic data

Type of participant	sex	Age Category				Total
			
		[20-35]	[35-50]	[50-65]	[65-...]	
Patient	m	1	1	0	0	2
	
	f	4	3	3	0	10

Family / Friend	m	0	2	1	0	3
	
	f	1	2	5	1	9

Care Provider	M	2	1	0	0	3
	
	f	3	6	0	0	9

Total		11	15	9	1	36

### Procedure

We sought the help of the heads of client programs (psychiatrists) to identify eligible participants (people with schizophrenia, affective, anxiety, or borderline personality disorders) and to obtain their cooperation in recruiting participants who met the inclusion criteria. The people with a mental health problem thus identified were contacted by the program heads to facilitate a preliminary contact and to get their permission for being contacted by the researcher. After the initial contact, the individual was asked to get in touch with the investigator to indicate whether or not he or she was interested in taking part and to confirm the participation of a family member or friend and of a care provider. The latter were also contacted to provide them with information on the research project and obtain their consent to take part in the study.

The research protocol was approved by the local medical ethics committee (CHUM, SL 06.055; HSCM, 2006-12-78; HLHL, 10-07). All participants received a written information sheet and consent form. The participants were thus able to give their free and informed consent to their participation in the study and the audio recording of the interviews. All recordings were kept under lock and key in a secure location throughout the collection and analysis of the data. The recordings will be destroyed by the investigator herself when the results are published.

### Conduct of the study

#### Data collection

Semi-structured interviews are a powerful tool and are deemed indispensable to an in-depth exploration of the participants' perspectives on the recovery process [22]. Three interview guides were used: one for people with a mental health problem, one for family members/friends, and one for care providers. The interview guides were based on empirical indicators from our earlier studies [3,4]. and the literature on the subject and drawn up by the investigators and their collaborators on the research team. The questions in interview guides are meant to encourage participants to clarify their thoughts with regard to their perspective on recovery and to explore more specific topics, such as personal conditions (e.g., sources of motivation, action strategies, personal resources), environmental conditions (e.g., interpersonal relations, social roles) and organizational conditions (e.g., accessibility of services, healthcare practices) that facilitate or impede recovery. It should be noted that the principal writer conducted the interviews.

Specially written forms were used to collect sociodemographic data for details on participant's sociodemographic data (n = 36), and the questionnaires were completed by the participants before their interview. The three interview guides had each been pre-tested with two people from the corresponding interview group, and minor alterations were consequently made. Guide topics focussed on the participants' perceptions of recovery and on the investigators' own research concerns. For example, questions for the participants with a mental illness dealt particularly with: (a) self-evaluations of their mental health; (b) perceptions of their recovery in biopsychosocial terms; and (c) the personal, environmental and organizational conditions that facilitated or impeded recovery [1,3,4,6]. The wording and presentation of the questions was flexible, and the investigator could modify them to adapt to the course of each interview. Throughout the study, the interviewer made field notes to record observations of the interview environment, factual occurrences, participant reactions, and personal impressions and reflections. The field notes were circulated among researchers to determine which data were most likely to be useful to our analyses.

Data were collected through 36 semi-structured interviews with the participants. Each interview lasting around 45-90 minutes, was digitally recorded and was transcribed and coded by the principal author and research coordinator. Immersion in the data resulted in the ongoing refinement of interview questions over the course of data collection and, consequently, in more accurate analysis of the data. The primary investigator combined her theoretical sensibility regarding this phenomenon with her clinical experience in mental health and continually correlated the study data with the relevant literature [1,4]. The content of this analysis and the categorization were discussed in depth with the research team.

### Data Analysis

The interviews were transcribed in full in order to carefully review the contents and, if necessary, further refine the interview questions and so ensure that the recovery process is properly delineated and understood. We transcribed the audiotape to conduct a qualitative content analysis using an approach based on the work of Miles and Huberman (2003) and Corin et al. (2005).

#### 1) The data analyses were carried out by the principal investigator and the research coordinator

**S**ignificant events, facts, and incidents were underlined in the text to help identify themes and key words. Themes such as relapse, strategy, motivation, and reference points were then grouped in individual tables to provide a comprehensive picture of each interview [23]. All the transcripts were then entered into *the Nonnumerical Unstructured Data Indexing, Searching and Theorizing *(NUD*IST) Vivo data-processing program to create a preliminary open-coding matrix. At this point, similar themes emerging from the interviews were organized by code. The data were then indexed, and the initial codes were grouped by properties or characteristics. The researchers examined the data and the relationships established between the different categories in order to carry out an intersubjective analysis as suggested by Miles and Huberman (2003) and verify the correctness and legitimacy of the procedures applied. Groupings were established among the categories when the investigators and collaborators judged the list of codes in the coding grids for the triads sufficiently exhaustive and meaningful. The codes associated with the categories formed in this way were grouped together into a single category. Individual analytic grids were then constructed for each participant and a trajectory grid developed that comprised the narratives of the three types of participant in the triad.

#### 2) Cross-analysis of qualitative data

By cross-analyzing between the data of 36 participants (12 triads analysis in 12 cases studies) [19], we were able to identify points of convergence and divergence with respect to a category and so describe the variations in the way recovery is perceived, interpreted and expressed in relation to the different contexts of interaction of each of the three participants (patient/family member/friend/care provider). Content cross-analysis was used to identify themes emerging from the interview data with 36 the participants or 12 triads. Finally, the set of main categories was established by grouping together subcategories with similar meaning (see Table 3 ).

**Table 3:** Summarizes reported dynamic context and relations influencing perspective of recovery of mental illness

Categories and subcategories	Dynamic Context	Relationship
Experiences before illness	Aptitudes Interests Plans for the future	Social network

Weakening factors	Family situation Disquiet	Relationship problems

Engulfment	Triggering factors Crisis	Involvement of family/friends

Living with the illness	Perception of the illness Turning point (trigger) Perception of state of health Wellness Management of symptoms Drug compliance Perception of recovery	Relations with self and others Changes Action strategies Social acceptance Support of family/friends, care providers Therapeutic alliance

Interactional barriers to recovery	Economic situation Family situation Isolation Rigidity of system of care	Pressure to perform Love relationships Stigmatization
Conditions conducive to recovery	Transformation Quality of services Points of reference Hope	Autonomy Relation to normalcy Life plans

The first column represents categories and subcategories. Columns 2 and 3 provide examples of dynamic context and relationships influencing the process of recovery of mental illness as reported in 36 interviews.

## Results

The objective of the study was to analyze the characteristic points of convergence and divergence between the three types of participants (patient, family member/friend, care provider) regarding the process of recovery of people with mental illness. Two main categories were formed from the analysis process of the content of 36 interviews with participants: 1) Revealing dynamic context, and (2) Relationship issues in a recovery process, furthering our understanding of such issues. The Table 3 uses these categories in presenting a summary of the dynamic context and relationship between the participants of each triad influencing perspective of recovery process as reported in the interviews.

To introduce to the significance of our findings with respect to the objective of the study, we briefly present four case studies which each associated with one of the four categories of psychiatric diagnosis: schizophrenia, affective, anxiety, or borderline personality disorder. For each case and for each actor, we describe the context and relationships: 1) the person with the mental illness; 2) the symptoms; 3) the crisis (triggering factors), and 4) the perspectives on recovery. The Table 4 summarizes this information about these four cases studies. These case studies will allow illustrating recovery from mental illness and showing how perceptions of the process vary depending on the participants' situation. In the research, a more detailed study of the 12 triads, each comprising a patient, a family member/friend and a care provider, was conducted with emphasis on points of convergence and divergence between their respective perspectives on the patients' recovery. Convergence means the three participants tend towards a similar perspective or the same goal with respect to a particular subject [19]. Divergence refers to a gap between the participants' perspectives (differing opinions) regarding a particular subject [19].

**Table 4:** Summarizes reported four case studies illustrated

Case	Diagnostic	Family/Friend	Care provider	Divergence
**1**. **Julie**, 37-year-old, 1^st ^hospitalization and diagnostic in 1990, no hospitalization in the last 5 years.	Schizophrenic disorder	**Emma**, 75-year-old, Julie's mother.	**Meg**, psychologist, 25 years practice.	Between Julie and her mother on internal and external aspect of recovery.

**2**. **Diana**, 52-year-old, 1^st ^diagnostic in 1990, 3 hospitalizations in the last 5 years.	Affective disorder	**Nancy**, 63-year-old, Friend.	**Eric**, nurse, 22 years of practice.	Between Nancy and Eric on the evaluation of Diana progress.

**3**. **Lilly**, 28-year-old, diagnostic in 2006, no hospitalization.	Anxiety disorder	**Mary**, 61-year-old, Lilly's mother.	**Sharon**, occupational therapist, 16 years of practice.	Between Mary and Sharon on the positive progress of Lilly.

**4**. **Eve**, 30-year-old, 1^st ^hospitalization and diagnostic in 2007, 2 hospitalizations in the last 5 years.	Borderline personality disorder	**Helen**, 56-year-old, Eve's mother.	**John**, support worker in a community organization, 3 years of practice.	Between Eve, Marie and John on the context.

In the following, we provide four case examples (among 12 cases studies) to illustrate the variations in the way recovery is perceived, interpreted and expressed in relation to the different contexts of interaction of each of the three participants (patient/family member/friend/care provider). To protect the informants' privacy, all their names have been changed.

### 1.1 How Julie (patient) perceives her recovery: experience of schizophrenia

Julie is a 37-year-old, middle-class woman. She had attended a private school. When she was about 20 years old, she had problems in her love life--"a heartbreaking time"-- and suffered great distress (L 18). She went three days without eating or sleeping and says she felt disconnected from reality: "*I was totally delirious. I wasn't there at all" *(L 24). In 1990, she was admitted to hospital and the psychiatrist diagnosed her as schizophrenia. She subsequently lived in a group home for two years. In 1998, she returned to and completed her studies. She now works in her field and feels better. "*I'm proud of what I'm doing now and what I've accomplished and that I can communicate and deal with people" (*L 189). She explains how her desire to pull through helped her to get over her problems: *My goal every afternoon was that I had to get out *[...] *I began to push myself towards something all the time" *(L 66). She makes the point that she has relationships outside her psychiatric social network and that she opens herself up to others. Julie has not been hospitalized in the past five years. She is working full time in her field and considers herself autonomous. Currently she has few symptoms and tries to enjoy herself by going to the theatre. She dreams of buying a house and is more open on the idea of having a love life. Julie considers she played an "active role" in her recovery.

### 1.2 How a member of Julie's family perceives her recovery: convergence and divergence

Emma, Julie's mother, is 75 years old and lives with Julie's father. Since her daughter was a child, Emma found her to be slow. She explained that Julie's father had drinking problems and that she encouraged her daughter to find things to do outside the home. During her very difficult adolescence, Julie fell in love with her karate teacher, a man 15 years her senior and a drug addict. After noting signs of disorganization, Emma was literally heartbroken to take her daughter back to the hospital. Emma acknowledges that her daughter is presently functioning well. Diverging from her daughter's perspective, Emma does not mention Julie's fighting spirit or role in her own recovery. Rather, she stresses the "outside" help her daughter received from care providers, medication, and the support Emma herself offered. There is a divergence too in some of the factors affecting recovery that Emma mentions, external variables that Julie does not refer to: economic insecurity and the pressures of stigmatization. It is noteworthy how much Emma's vision diverges from her daughter's with regard to the active role Julie sees herself as having played in her recovery.

### 1.3 How Julie's care provider perceives her recovery: convergence and divergence

Meg, a psychologist who has treated Julie, says that Julie's path has not been linear. Over the course of a number of relapses and admissions to hospital, Julie was diagnosed with schizoaffective disorder. Meg has met Julie's parents a number of times and notes the difficult relationship between Julie and her overprotective mother.

"*I don't criticize the mother at all. I think she's learned to adapt. But when your child becomes ill I *[...] *think there's a tendency to become overprotective *[...] *and the overprotectiveness went on for many years*" *(Meg, L 106)*.

According to Meg, Julie has suffered a number of bereavements related to her illness (e.g., the loss of her home, her spouse, her plans for an education, her job). After her stays in hospital, she went back to live with her mother. Meg said that the hardships Julie suffered are rarely discussed with her mother. She always liked to have project to be involved in. She was given certain responsibilities in the group home, sitting on the Board of Directors, for example. She completed her community-college level studies. More over, Meg says: "*Her family was always there for her and was never ashamed of her illness" *(L 56). She underscores how important Julie's fighting spirit was to her recovery, a spirit characterized by openness to others and a capacity to seek out the resources she needs. She underscores Julie's active role: she is a member of the board of directors of a community organization. Through her social involvement, Julie has succeeded in constructing another identity, different from that of the "sick person" she saw herself as at the onset of her illness.

### 2.1 How Diana (patient) perceives her recovery: experience of affective disorder

Diana is 52 years old. She told us that she has always felt unhappy, even as a child. She worked as a school-bus driver for 16 years and loved her job. She had long felt that her relationships with others and her love life were very intense. She did so of her own accord and in 1990 was first diagnosed with manic depression; she felt relieved. She took lithium for four years, which stabilized her mood. Over the last 5 years, Diana has been hospitalized three times. She now has access to a transition house where she lives alone in a studio apartment. She has been followed by a psychiatrist, a social worker and several support workers in community organizations. Diana feels she is better and that she will soon be able to go back to work. "*I feel like I've recovered and a lot better now" *(L 6). She still needs the support of her care providers. She has re-established contact with her family. She considers that she is recovering because she is able to find the discipline to go to her appointments and her craft and photography workshops and to take her medication. She also uses breathing techniques and prayer.

### 2.2 How Diana's friend perceives her recovery: convergence and divergence

Nancy is the person Diana identified as a "significant friend" for the purposes of our study. Nancy underscores the bereavements Diana has had to suffer due to her illness: the loss of her job, her home and the pets she loved. Shaken by a stay in hospital, Diana said she was "sick" of being on the street and of having legal problems. Nancy says that Diana's mother is grappling with affective disorder - a point Diana does not mention. According to Nancy, Diana is presently very fragile as regards her alcohol dependency, despite her claims that she has completely gotten over it. She smoked some pot recently under the influence of her brother. In Nancy's opinion, Diana is in a process of recovery but has not recovered: "*She still has a lot of work to do" *(L 134). She has renewed contact with members of her family, who also have dependency problems. Her desire to live is currently stronger than her desire to die. Diana has enormous potential because of her sociability and fighting spirit.

### 2.3 How Diana's care provider perceives her recovery: convergence and divergence

Eric is a nurse and has been following Diana for one and a half years, seeing her once a month. He feels Diana currently has the means to reorganize her life. In contrast to Nancy, he maintains she has stopped drinking: "*It's been totally worked out. She's never touched it again*" (L 126). Diana is strongly motivated to get better. She is smiling more and seems to have a greater sense of well-being. She has renewed contact with her family. In Eric's opinion, Diana contributes to her recovery by being very involved in her treatment and every aspect of her follow-up. He underscores the importance to her recovery of her acceptance of the illness and the medication and of the trust relationship she established with her support worker.

### 3.1 How Lilly (patient) perceives her recovery: experience of anxiety disorder

Lilly is 28 years old. She has always been very successful in the various aspects of her life. She is a figure skater but had a hard time managing her nerves in competition. She has a bachelor's and a master's degree. She always did very well at school but feels that she had to put more time into her studies than other people did; she was unable to concentrate. She suffered a crisis when she started to work full time, but has never been hospitalized. She was afraid she would not close the door properly, so refrained from going out. In 2006, she saw two or three psychologists who told her she had an obsessive compulsive disorder (OCD). She felt relieved by the diagnosis one year after the onset of the problem. At the same time, her mother told her that she too had suffered from OCD when she was young. This information upset her because she had never felt that her mother gave her any support.

"*Why didn't she ever support me when she knows what it is *[...] *I never felt she understood me or even supported me*" (L 128).

Lilly now works in a dollar store, but feels a great deal of pressure from her parents to find a better job. She says she has always been motivated to make plans. She is gradually learning to enjoy herself and relax. She feels that she is playing a role in her recovery and that the plans she makes, her reading about the disorder and the different methods she uses (yoga, walking) help her feel better. She feels she is in recovery because she has made a great deal of progress since being diagnosed, although there are still days she feels fatigued and irritable and has trouble concentrating. She gets out of the house more (something that used to make her anxious) and has new friends.

### 3.2 How Lilly's mother perceives her recovery: convergence and divergence

Mary, Lilly's mother, is 61 years old. She lives with Lilly's father, who owns a company. The couple work long hours there. Lilly's father was a high-achiever at work, a fact that put pressure on Lilly. Mary remembers that she excelled in her studies at school and in figure skating. Mary is aware of the difficulties her daughter has gone through, but now she thinks that Lilly is "vegetating".

"*She got through her studies by the skin of her teeth *[...] *but since finishing, she hasn't used her degrees. She's been vegetating for 5 years now" *(L 66).

Though she has not admitted it to her daughter, Mary hopes she has no children because of the risk of passing on the illness. There is a divergence in perspectives in that Mary does not see Lilly's role in her recovery in the same way her daughter does. In her opinion, her daughter is better because of the medical follow-up, the passage of time and the breach in her isolation. In contrast to Lilly, Mary does not acknowledge the influence of any active role by her daughter in her own recovery. She thinks her daughter must feel guilty for not functioning normally and must face prejudices about mental illness. Lilly does not mention either issue in her interview.

### 3.3 How Lilly's care provider perceives her recovery: convergence and divergence

Sharon is an occupational therapist who has followed Lilly for one year after she was evaluated by a family physician and a psychiatrist. She explains how grave the prognosis was at the onset of the illness, a point neither Lilly nor her mother makes. She no longer showered because she was afraid of contamination. She suffered from memory and concentration problems, intrusive rituals and very high anxiety. Lilly called her problem her "jail" because she had no freedom of action, and she wanted to pull herself out of it. Sharon points out Lilly's greater autonomy about her disorder and acknowledges her role in her recovery. She notes the reduction in symptoms and the progress Lilly has made, pointing out her greater sense of well-being. She notes that Lilly's relationship with her parents is very troubled since they do not express their emotions.

"*The relationship with her parents was very cold. For them, life is meant for work and being successful" *(L 81).

Lilly is in the process of rebuilding herself, Sharon says. *She supported Lilly in her choice of a job that is not demanding on a cognitive level so that she could re-establish contact with people*. Sharon notes that Lilly's medication seems to be working and that she manages her symptoms better.

### 4.1 How Eve perceives her recovery: experience of borderline personality disorder

Eve is 30 years old and works in the dispensary in a pharmacy. Even before her diagnosis, she noticed she had been having problems of impulsiveness, of managing her emotions, of loss of interest, and of periods of depression since she was 17. In early adulthood she also noticed problems with concentration and in her love life. She had many conflicts with her parents, who did not understand the situation and said she was lazy. Gradually, she stopped doing any housework, cut herself off from people and stopped paying her bills. In March 2007, she attempted suicide at work, a pharmacy where she was a dispensary technician. "*She sought help in a crisis centre and was also followed at an outpatient clinic at the same time*". Her diagnosis of borderline personality disorder took a weight off her shoulders, especially because people acknowledged she needed help. She currently feels she is better and that she is happier because she is more stable emotionally. She is able to assert her needs better and validate her perceptions with others. She feels people have rallied around her and uses the help proffered by other people (particularly her support worker). Still, she feels that she has to play the primary role in her recovery.

### 4.2 How Eve's mother perceives her recovery: convergence and divergence

Helen, Eve's mother, is a 56 year-old primary-school teacher. Helen says that Eve had dreamed of working in politics and had very good marks at school. Her problems began when she was about 17, when she became very passive. Helen recently found out that her daughter had been sexually abused by her brother during adolescence.

*"I sent for her brother and he admitted that some minor things had happened but that they weren't serious, but Eve said: "It was serious because it left its mark on me" *(L 44).

Since she no longer studied, her parents insisted she get a job, so she started work at the pharmacy. Eve had a difficult relationship with her boyfriend, whom she moved in with when she was 21. Helen interprets the suicide attempt as an attempt to find help. Helen helped her daughter buy a condominium, and Eve takes care of it and pays the bills. Eve has trouble maintaining stable friendships and love relationships. Helen thinks it is a pity her daughter did not pursue her studies. She says Eve has a tendency to dream about life rather than live it. She makes the point that the support she gives her daughter (particularly the financial help she gives her by contributing to the mortgage for the condo) is helping her daughter get better. Helen acknowledges changes in Eve's condition: She is more open to others. She is better when she is on her own. She manages her emotions better. She is less aggressive. She can look at things more clearly. She has friends and keeps them. Relations with her daughter are simpler. At the day hospital, they laid a lot of emphasis on her daughter's strengths, which enhanced her self-esteem.

### 4.3 How Eve's care provider perceives her recovery: convergence and divergence

John is a support worker in a community organization for people with mental-health problems. He told us that Eve was reportedly sexually abused as a child and as an adolescent by her brother and her grandfather. John says:

"*Yes, there was an assault by her grandfather that happened only once *[...] *it stopped right away. There were also assaults by her brother over a longer period of time" *(L 30).

When she attempted suicide, she was at a point of great suffering in her life. She felt very much alone. She tried to distance herself from her parents and seek some autonomy, but she realized doing that did not assuage her suffering. With counselling, she began to recognize her strengths and rebuild a more positive self-image. She has started painting and drawing again and to take "small steps" to gradually becoming autonomous. She is open about her homosexuality with her counsellor; neither Eve nor Ellen mentioned this point in their interview. She has gone through a process of mourning, which, John thinks, explains her recovery. "*I think she was very motivated to pull through and that she was fed up with the suffering" *(L 68). John also underscores the importance of the stability of the intervention.

#### Enhancing understanding of the recovery process

##### 1. Revealing dynamic context

By collecting data through individual interviews with each participant in the triad of patient, friend/family member and care provider, we are able to document in detail the context and dynamics of the recovery of a person with a mental disorder. The family members/friends and care providers bring to light very important events--which the patients do not mention--that provide for a better understanding of the patients' background. For example, Eve did not mention the sexual abuse by her brother, but her mother did. More strikingly, her counsellor provided further information, saying Eve had been abused--not just once but repeatedly--by her brother and once by her grandfather as well. The sexual abuse helps put the intensity of Eve's childhood and adolescence into context and enlightens us about her recovery process. Triangulating the perspectives of each participant on the individual's recovery yields much finer and more detailed pictures and interpretations of events and thus helps make a more complete analysis of the important issues in the patient's recovery [22,23]. Indeed, a person's life trajectory is generally expressed in a complex network of relationships that is revealed not only in tensions (and sometimes traumas), but also in various forms of support and encouragement. What is more interesting is that triangulating the perspectives (of patients, family members/friends, and care providers) reveals relationship issues that would otherwise remain undiscovered.

##### 2. Relationship issues in a recovery process

A part from helping document the life context of the people experiencing recovery, the triads enable us to bring to light data on points of divergence. Julie's (case 1) shows clearly how, from the standpoint of the client, recovery is experienced as an inner struggle involving her entire being: "*I still had this sort of life inside me*" (L 70). Her mother, Emma, linked Julie's recovery to--among other things--her external resources and support: the psychological follow-up and compliance with medication. In Lilly's (case 3) there is a striking difference between her and her mother with regard to Lilly's role in her recovery. Lilly spoke about nervousness, problems concentrating, about her suffering and her relief when she received her diagnosis and felt that, at last, people understood her. Helen, for her part, does not acknowledge her daughter's power in attaining her greater well-being. She stresses Lilly's inactivity and need to join the job market, for, in Helen's view, recovery involves first and foremost concrete action: "*You've got to find a job. You've got to make something of your life*" (L 52). The patients pointed up their strength of will, their combative spirit and the efforts they made in order to feel better despite their disorder. For them, recovery is a feeling of inner well-being. For family members and friends, recovery is perceived in terms of observable signs, such as the capacity to go out, to establish contact with other people, and to be autonomous (be independent, pay bills, hold a job).

They lay less stress on the individual's inner strengths. While not necessarily disregarding the "appearance" of greater well-being, they link it to socially accepted criteria. Interestingly, in this triangulation of perspectives, the care providers all point out the patients' motivation to transcend the symptoms of their disorder and highlight their strengths. Quite often, neither patients nor their friend/family member describes their strengths so clearly. We consider this an important finding since it indicates that the trust relationship essential to the establishment of a therapeutic alliance has been created. In cases in which there are palpable tensions between patients and the people close to them, the bond with the care provider assumes major importance. In certain contexts, care providers play an important role in cases in which there is tension between patients and their family and friends. A psychologist pointed out that some movement is required on the part of family members and friends supporting the patient; they have to question some of their own expectations.

"*It's not easy in relations with family and friends *[...] *you also have to make them question themselves with regard to of their expectations *[...] *their goal is not necessarily the same as the patient's. So they have to move a bit too. So you've got to lead the parents to question themselves without losing the trust relationship because it's important*" (care provider, L 156).

To sum up, by comparing the different perspectives we were able to uncover a more extensive background of relationships than if we had only the interview with the person with the mental health problem to work with. We were thus able to grasp more of the complexity of the relationships that are part of the context of recovery.

## Discussion

The differing perspectives of patients, their significant family members or friends and care providers have rarely been compared; nor has the way they may affect and structure recovery-oriented clinical interventions been widely explored [19,20,24]. Our findings underscore the impact of context and relationships on recovery and hence the differing perspectives that stem from the experience and expertise of the people directly concerned. Our results indicate the need for a careful consideration of the clinical implications of what the different actors perceive, interpret and express as a function of their frame of reference and the context of their relationship.

Several studies reserved a place for life stories, narratives testifying to people's experiences, the obstacles they have encountered, and the things that have helped them [25-28]. At the same time, research based on long-term follow-up of people with mental disorders shows that the progression of such conditions varies more widely than thought [29-31]. Attention has shifted to the need to consider what people bring with them given their situation as well as the meaning they give to their life and the projects they wish to accomplish [32]. The idea of recovery, as it has been developed in the literature, draws attention to the need for a broader set of objectives and for support for people as they pursue their own projects and strategies [33-37].

The involvement of patients and family member's or friends in planning mental-health-care services reflects the concern to play a central role in the process. However, their mere inclusion in public forums of discussion of care and services does not necessarily mean they will be listened to or participate in any meaningful way [38]. We have therefore adopted a new methodological approach to understanding the recovery process by using data drawn from three sources: people with a mental illness, their family member's or friends, and health professionals. People with a mental illness are obviously the most qualified to discuss their own perceptions and day-to-day experiences. Close relatives and friends are "actor-observers" and thus also a source of that they ought direct accounts of a patient's history. Health-care professionals and care providers, too, have a special relationship with their patients; they are present in situations that give them insight into a patient's progress, potential, and follow-up, and they are best placed to consolidate a patient's resources. They are also often witness to significant breakthroughs and outcomes that surpass what would be considered normal improvements or stabilization in the condition of a person with a mental disorder [4].

We think it is important to continue investigation of the recovery process in mental health through triads comprised of a patient, a significant family member or friend and a significant care provider. The purpose of eliciting the differences in the actors' perceptions is to develop a clinical practice that will provide individuals with real support and backing in their recovery. Interventions must thus take into account areas of divergence and convergence between the perspectives of the three actors with regard to the way people recover, to what helps and what hampers recovery. Our research calls on care providers who wish to promote the recovery of individuals with mental health problems not to ignore the role that patients, their relatives or friends and their care providers can play in the process. Studies often focus on conditions that are, at first sight, ideally conducive to recovery but do not always consider factors that hinder it. However, the hindrances are the real challenge that people who are trying to recover must face.

Many studies deal with what recovery means in general terms for clients, care providers and policy makers, that is the stakeholders who receive, provide and plan mental-health-care services [39-45]. Our research would seem to complement these studies, which stress the importance of considering the notion of subjectivity associated with recovery and of examining a variety of information sources to better understand the complex dynamics of the process. The studies by Noiseux (2004), Noiseux and Ricard (2008), and Noiseux et al. (2009) take another approach, basing analysis on triads made up of the individual concerned, one of his or her family members or friends, and one of his or her care providers. From this standpoint, these studies are more rooted in "close intervention." They not only explore the respective perceptions of the three actors in the triad, but also involve a care provider and friend or family member designated by the individual as *significant *to him or her. This approach accords with the recovery-oriented perspective in that it seeks to truly take into account the individuals' viewpoint, with the aim, most especially, of empowering them, of giving them back power over their own life. These two research approaches seem to us to illustrate the difference between a research project about recovery and one that draws its inspiration directly from the concept of recovery to provide practical guidance for interventions.

The individual in recovery is often placed at the centre of considerations of the phenomenon. However, the way in which his perspective is related to mental-health stakeholders will affect the way we understand the phenomenon and intervene on it. For example, in the "vertical" vision such people tend to be depicted as "consumers" with respect to the orientation, quality and organization of services. The point of view of the individual in recovery is thus related to that of the clinicians and policy makers and is accordingly contemplated from the broader perspective of "a recovery-oriented health-care system."

The vision of recovery in our study may be described as a "horizontal" one. It involves patients, family members or friends, and care providers, and the emphasis is on individuals in recovery in terms of the dynamics of their context and how they relate to themselves and others. The process of recovery is considered to be a nonlinear trajectory experienced by an individual [33-37,39,40]. The focus is on the capacities for feeling, thought and action of the actors most directly concerned and on the bonds or relations between them. Because the aim in adopting this "horizontal" vision is to affect the therapeutic process, the "patient" with his history, suffering, strengths, and weaknesses is placed centre stage [26,33-36]. Despite the differences, the two visions are complementary, for the individual in recovery is both a "consumer" and a "patient." The literature demonstrates the clear will and enthusiasm of policy makers and mental-health departments to develop recovery-oriented care and services. This enthusiasm is legitimated by political pressures that are exerted as well as by the actions of groupings of different categories of actors aimed at to achieving the same aim. However, such enthusiasm is also liable to give rise to a polarization of resources rather than to situations conducive to negotiation and alliances between the actors directly concerned (patient, family member or friend, care provider).

We think it is important to continue qualitative research into the recovery process [46]. By making it possible to elicit the perspectives of actors based on a "vertical" or "horizontal" vision, qualitative research has given us access to knowledge of the dilemmas and issues people face during recovery *from the inside *[20,46,47]. Studies based on a notion of recovery in mental health point to the necessity of a more complex understanding of the meaning attributed to mental-health problems [24]. This meaning constructed in a particular context [18,48]. Account must also be taken of the differences between the interpretations formulated by patients, their friends or family members and care providers [49].

### Implications for research and clinical benefits

In terms of advancing knowledge in the field of mental health, the comparison of viewpoints made possible by constructing the triads yields benefits for analysis in that the quality and breadth of the new information gathered casts light on the complexity of the context of recovery. The study's heuristic value lies too in helping improve understanding of the dynamics of the mechanisms involved in the phenomenon under study: patients are not alone, but face a singular context: family members, friends and clinicians who are invested with the power and authority to define and legitimate certain social norms [19,20]. In fact, the results reveal that recovery tends to take different shapes depending on whether it is a process one experiences or a result one expects; both views, of course, have some legitimacy. The issue here is to create a clinical space for discussion and negotiation in order to offer real backing to people in their recovery and support their family members and friends during the process.

In clinical terms, if one considers that the notion of recovery varies with the person involved (patient, family member/friend, care provider), it is plausible too that it varies with the different symptoms associated with particular mental disorders. Schizophrenia involves one or several sporadic psychotic episodes and spectacular symptoms, while borderline personality disorder relates to a personality structure that has apparently taken shape in a gradual process spread over many years [50]. With this point in mind, our analyses seem to reveal points of divergence, particularly between these two diagnoses, with respect to the circumscribed nature of the notion of recovery for schizophrenia and the diffuse character of recovery in borderline personality disorder.

### Criteria of scientific rigour in qualitative research

Like other qualitative research methods, case study meets the criteria of scientific rigour: credibility, transferability, and that conclusion or propositions respect the data that has been collected [51,52]. To ensure credibility internal consistency, and reliability. **Credibility: **This criterion entails data authenticity, the research-team investigators and collaborators were returned continually to the empirical data to back up the organization and interpretation of the data. More specifically, field notes were made and the data transcribed in full as the study progresses, thus increasing its credibility. In addition, continuous rereading of the transcripts, constant revision of the coding by comparing empirical data and meticulous analysis of the data will enhance the authenticity of the findings. **Transferability: **Respect for the principles of theoretical sampling and empirical data saturation make transferred of the conclusions to settings similar to the one under investigation possible. The detailed description of the settings in which the research has conducted help determines whether or not the conclusions can be transferred. **Internal consistency: **This criterion relates to the quality of the description of the analytic process. Triangulation with different data-collection tools makes it possible to track and follow the logic of decisions that are made his criterion can be met by reference to the interviews, the field notes, the systematic and rigorous organization of the data, and the open-coding grids of the recovery process. **Reliability: **This criterion was respected by virtue of providing a transparent explanation of the methodology used in analyzing the data, a detailed description of the people providing the data.

### Limitations of the study

The social desirability bias, defined as the tendency of participants to give socially acceptable answers to questions they are asked, may limit the breadth of data collected and, consequently, the depth of the analysis. The investigators took a series of measures aimed at achieving the best possible control for this limitation. For example, the investigator insisted on the establishment of a trust relationship with each of the participants throughout the course of the interviews. She made clear at the outset and throughout each interview that there were neither good nor bad answers to the questions she asked. Case studies conducted a constructivist paradigm [15] involve data interpretation by the people doing the research. In fact, the results they obtain depend on their creativity and conceptual skill [53]. The investigators and their collaborators recognize that, though based on a rigorous approach to the organization and analysis of the data, the study of the process of recovery is a human construct.

## Conclusion

In conclusion, this qualitative study comparing the perspectives of three types of actors (patients, family members/friends and care providers) suggests that the recovery process depends on constructing meaning around mental illness experiences and is based on each individual's dynamic context (e.g. social network, relationship), life experiences and other social determinants (e.g., symptoms, environment). The findings from this study add to our existing knowledge about the determinants of recovery for persons utilizing public mental health services in Montreal, Canada. The others parts of this study will continue the analysis with special reference to the impact of the notion of pluralism (dynamic context). The aim is to develop a model of recovery in mental illness and identify strong empirical indicators that can be validated and applied to tailored interventions in order to improve mental health practices and services.

## Competing interests

The authors declare that they have no competing interests.

## Authors' contributions

All the authors read and approved the final manuscript and take responsibility for appropriate portions of the content. SN contributed substantially to the concept and design, data acquisition, analysis and interpretation, and general supervision of the research group. DSCT made important contributions to the critical revision of the manuscript in terms of major intellectual content and analysis and interpretation of data. EC was a major contributor to the cross analysis and interpretation of data. PLSH was involved in the cross analysis and interpretation of data. RM made important contributions to the critical revision of the manuscript in terms of major intellectual and clinical content. CL was involved in obtaining approval for the research project from the CHRTR Science and Ethics Committee and in the data analysis. Finally, DF, LV, FG were involved in the selection of participants and the interpretation of data.

## Pre-publication history

The pre-publication history for this paper can be accessed here:

http://www.biomedcentral.com/1472-6963/10/161/prepub
